# Riding the waves of the intercalated disc of the heart

**DOI:** 10.1007/s12551-018-0438-z

**Published:** 2018-07-09

**Authors:** Pauline M. Bennett

**Affiliations:** The Randall Centre for Cell and Molecular Biophysics, School of Basic and Medical Biosciences, New Hunt’s House, Guy’s Campus, Kings College London, London, SE1 1UL UK

**Keywords:** Cardiomyocytes, Intercalated disc, Spectrin, Protein 4.1, Transitional junction, Cardiomyopathy

## Abstract

Cardiomyocytes interact with each other at their ends through the specialised membrane complex, the intercalated disck (ID). It is a fascinating structure. It allows cardiomyocytes to interact with several neighbouring cells, thereby allowing the complex structure of the heart to develop. It acts as tension transducer, structural prop, and multi signalling domain as well as a regulator of growth. It achieves its many functions through a number of specialised domains and intercellular junctions associated with its complex folded membrane. This review outlines the results of some 20 years of fascination with the ups and downs of the ID. These include locating the spectrin-associated membrane cytoskeleton in the ID and investigating the role of Protein 4.1R in calcium signalling; structural studies of the relationship of the ID to myofibrils, sarcoplasmic reticulum and mitochondria and, finally, consideration of the role of the ID in cardiomyocyte growth and heart disease.

## Introduction

In the early 2000s, I joined a group headed by Dr. Jenny Fordham to work on a new project looking at the spectrin-associated membrane cytoskeleton in the heart. One of the first experiments was to look at the localisation of αll spectrin in cardiomyocytes by immuno-fluorescence. As well as showing up at the Z-disc level throughout the cardiomyocyte, it was very strongly present at the intercalated disc (ID) (Bennett et al. 2004). This observation was the start of a continued affair with the intricacies of the ID. These studies have ranged from the role of spectrin and protein 4.1R in signalling domains at the ID, particularly calcium signalling, structural studies of the relationship of the ID to myofibrils, sarcoplasmic reticulum (SR) and mitochondria, to a broader consideration of the role of the ID in cardiomyocyte growth and heart disease.

**Fig. 1:**
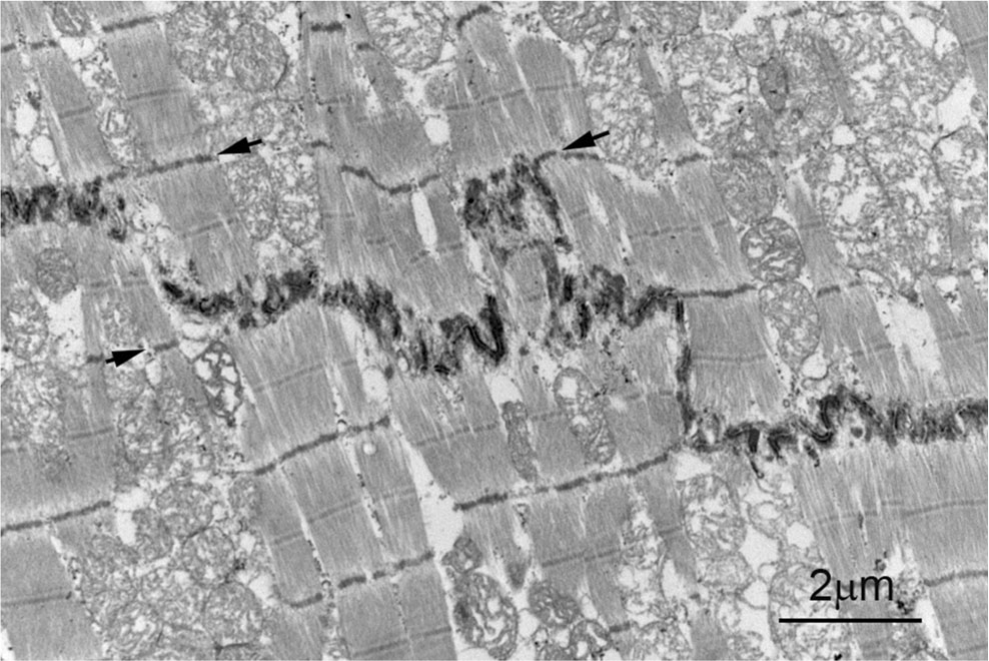
Electron micrograph of a section of the left ventricle of human heart. The ends of two cardiomyocytes meet at the stepped ID, the dark line of stain that crosses the image. The plicate transverse treads and the narrower longitudinal risers can be seen. Arrows indicate Z-discs near steps, which align axially with one edge of the ID. Mitochondria are poorly preserved because of ice damage during tissue storage

**Fig. 2:**
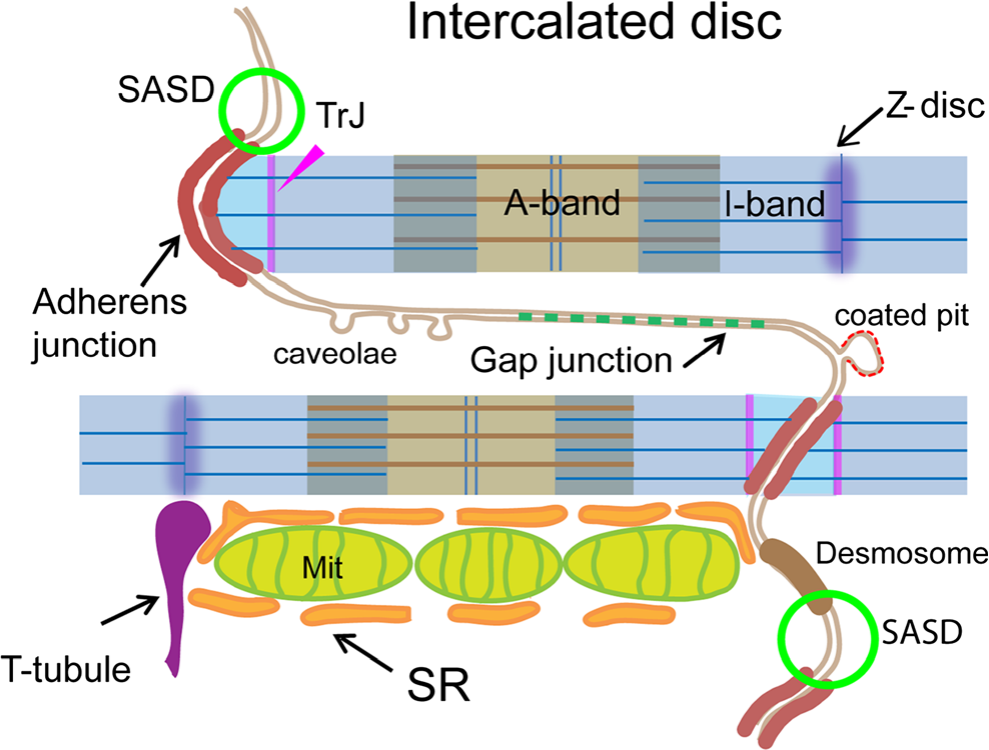
Diagram to show the domains associated with the ID. A step in the ID with two transverse plicate steps and a longitudinal riser are shown. The membrane accommodates the three types of intercellular junctions, the adherens junction, the desmosome and the gap junction. On the regions of uncoated membrane are found caveolae and coated pits as well as, at the top of the membrane folds, the spectrin-associated signalling domains (SASD) (green circles). The thin filaments from the last sarcomere of the myofibrils insert in the AJ passing through a transitional junction (TrJ—pink line) rich in Z-disc proteins. Mitochondria (Mit) columns run parallel to the myofibrils finishing at the edge of the ID. SR is sandwiched between mitochondria and ID membrane

Cardiomyocytes interact with each other at their ends through the specialised membrane complex, the ID. Each cell can overlap with several others creating a three- dimensional organisation. Many wonderful electron microscope studies have been carried out, (see e.g. (Fawcett and McNutt 1969; Forbes and Sperelakis 1985). Figure. 1 shows an electron micrograph of a longitudinal section of an ID in human heart. It illustrates the stepped structure with narrow longitudinal risers and transverse steps. In the transverse steps, the membrane is very plicate rather in the way of an egg box so that in longitudinal section it appears as folds and, transversely, as loops or circles of membrane. This stepped shape is closely correlated with the sarcomere structure of the fibrils. The risers are an integral multiple of the sarcomere length, usually one, and the peaks of the folds correspond to the level of Z-discs at the steps so that sarcomeres in neighbouring cardiomyocytes are displaced axially by the width of the ID (Figs. 1 and 2). There are three well- characterised types of cell-cell junction within the ID; the adherens junction (AJ) where the thin filaments from the myofibrils invest and where the active tension is transmitted from cell to cell; the desmosome which acts as a spot weld to mechanically keep the cells together and anchors the cytoskeletal desmin filaments, and the gap junction where the electrical signal is transmitted. In addition, there are regions in the ID where there are no intercellular junctions and which are of no obvious function most notably on the risers and at the top of the folds. Here, the membrane separation is variable, but there is no extracellular matrix such as that associated with the lateral membrane of the cardiomyocytes. In these regions, caveolae and coated pits are often seen indicating the active role of the ID (Bennett 2015; Forbes and Sperelakis 1985). In addition, in these bare regions at the top of the ID folds, we found spectrin to be located (Bennett et al. 2006; Wilson et al. 2014) (Fig. 2).

## Spectrin-associated signalling domain at the ID

The discovery of αll spectrin at the top of the folds indicated that it is the spectrin-associated cytoskeleton that supports the membrane here. This correlates with the absence of dystrophin at the ID (Stevenson et al. 2005). The spectrin complex is generally present in several locations in the heart but several components are strongly represented at the ID, such as β spectrin, protein 4.1R and 4.1 N , ankyrin G (AnkG) and Band 3 (AE1) (Isayama et al. 1993; Messina and Lemanski 1989; Mohler et al. 2004; Moura Lima et al. 2003; Pinder et al. 2012). Several signalling proteins have been associated with this complex lending support to the presence of a localised spectrin- associated signalling domain (SASD) at the ID (Fig. 2). An example of this is the binding of Mena/VASP to αll spectrin to regulate cytoplasmic active actin complexes, the absence of which leads to dilated cardiomyopathy (DCM) (Benz et al. 2013).

Another signalling protein, the sodium voltage channel Nav1.5, is important in electrical signalling and is thought to be vital for ephaptic charge propagation in the heart (see Manring et al. this volume). Absence of competent Nav1.5 leads to severe arrhythmias and heart failure. Mohler and his colleagues have identified a direct interaction between Nav1.5 and AnkG (Lowe et al. 2008) and the conditional knock- out (KO) of AnkG in mice leads to the loss of Nav1.5 specifically at the ID (Makara et al. 2014). Protein 4.1R also appears to affect the localisation of Nav1.5 in the heart. In our studies of a 4.1R KO mouse where only a small fragment of the protein is expressed, we found that the level of Nav1.5 was significantly reduced (Baines et al. 2009; Pinder et al. 2012; Stagg et al. 2008). Physiologically, there were defects in calcium signalling leading to bradycardia. However, there were no changes in levels of the sodium calcium exchanger NCX, or Na^+^/Ca^+^ ATPase (Stagg et al. 2008), although by immunofluorescence, they both colocalise at the ID with protein 4.1R and not with the proteins of the structural domains, such as connexin 43 and β- catenin (Pinder et al. 2012). While we did not determine which factors were directly involved in the protein 4.1R KO behaviour, it is known that protein 4.1R and the spectrin complex in general, can act as an interaction hub for a wide variety of membrane signalling processes (Baines 2010; Baines et al. 2014). Many of these overlap the more than 200 proteins which have been associated with the ID (Estigoy et al. 2009).

## The ID relationship with myofibrils; the transitional junction

In the original experiments to locate spectrin in the cardiomyocyte, we used myofibrillar proteins as controls and found that Z-disc proteins, in particular Z-disc titin, were also split across the ID with the same spacing as the spectrin (Bennett et al. 2006). This prompted a consideration of a fascinating aspect of the relationship between myofibril and ID. That is, that the ordered sarcomeric structure is very well maintained right up to the edge of the ID. However, there is no dense Z-disc seen at the ID end of the fibrils, rather the thin filaments run from the last half sarcomere to insert their barbed end into the AJ plaque at the ID (Forbes and Sperelakis 1985; Yamaguchi et al. 1988). Since the ID membrane at the AJ is not transverse, but angled, these filaments are all of different length. If titin were anchored to the end of the thin filaments at the ID as at the Z-disc, its elastic spring would exert different forces on the thick filaments of the A-band and lead to disorder. However, the splitting of the titin antibody label across the ID argued for a spot along the thin filaments, where the Z-disc proteins are anchored which is equivalent to a Z-disc. We called this the transitional junction (TrJ) (Bennett et al. 2006) (Fig. 2). It allows titin to be attached at a constant distance from the ends of the thick filaments and the order of the final sarcomere to be maintained. The TrJ contains Z-disc proteins, titin, α actinin and ZASP but not telethonin, FATZ or CapZ (Bennett 2012; Bennett et al. 2006). It is essentially an immature Z-disc as further indicated by the presence of Nrap (Luo et al. 1999; Zhang et al. 2001). Beyond the TrJ into the ID folds, the evidence so far suggests a different actin, possible β or γ actin with no sarcomeric thin filament proteins (Bennett et al. 2006; Benz et al. 2013). Considering that the thin filaments from ID to A-band are continuous, it is not clear how this organisation comes about. A similar mechanism might be involved in the myofibril to tendon transition in the myotendonous junction in skeletal muscle.

The position of the spectrin- rich domain in close proximity to the edge of the fibril at the TrJ level suggests an interaction between the two, which constrains the tops of the folds to this position. This may be mediated by filamin C, which also exhibits the split distribution across the ID (van der Ven et al. 2000)

## The ID relationship with mitochondria and SR

The energy requirement of the cardiomyocyte means that a large volume of the cell is filled with mitochondria. In transverse section, this is of the order of ~ 30% of the area of the cell. Mitochondria are found in columns that run parallel to the myofibrils. The columns reach as far as the ID but, like the fibrils, rarely penetrate beyond the tops of the folds (Bennett et al. 2016) (Fig. 2). The columns are intersected by t-tubules at the Z-disc. SR wraps around myofibrils and mitochondria and interleaves itself between t-tubule and mitochondria where it forms dyads. Electron tomography of the ID has revealed the complex shape of the ID membrane and also its relationship with mitochondria and SR (Bennett et al. 2016; Leo-Macias et al. 2015; Pinali et al. 2015). We find that in the absence of t-tubules at the ID, SR forms dyad attachments to the cell membrane towards the tops of the ID folds, which are frequently sandwiched between membrane and the mitochondria (Fig. 2). The SR rarely reaches further into the ID folds. These studies show the intimate relationship between mitochondria and SR at the ID and place them close to both the TrJ and the spectrin-associated signalling domain.

## The ID and cardiomyocyte growth

The width of the ID, that is, the amplitude of the folds is variable. Locally, it is quite uniform but it can vary considerably in different areas of the heart, from 200 nm to 2 μm with an average spacing of ~ 500 nm (Wilson et al. 2014). It is notable that the size rarely exceeds 2 μm, the sarcomere length. Increasing the ID size allows cells to grow gradually by a few per cent. Further growth requires new sarcomeres, and it has been suggested that they can be inserted at the ID (Bennett 2015; Wilson et al. 2014; Yoshida et al. 2010). Yoshida et al. (2010) have shown characteristic changes in ID morphology concomitant with sarcomere addition. We also have found suggestive evidence in electron micrographs. In some cases, individual thick filaments can be seen in long folds where there is still a TrJ and no visible Z-disc. Elsewhere, whole sarcomeres are enclosed within a fold (Bennett 2015; Wilson et al. 2014). The model proposed is that new thick filaments can be formed or inserted into long folds. When there are sufficient of them, the TrJ is stimulated to become a mature Z-disc and a new sarcomere is formed (Bennett 2015; Wilson et al. 2014). Mitochondria growth will then occur to keep pace with new sarcomere formation (Bennett et al. 2016).

## Disease and the ID

There are many accounts of changes in the ID associated with heart disease (see Bennett 2015)). In the extreme case of arrhythmic right ventricle cardiomyopathy, loss of or mutation in desmosomal proteins leads to the replacement of cardiomyocytes by adipose tissue (Sheikh et al. 2009). In general, the changes at the ID caused by disease include increased size and disorder. It has been suggested that DCM, in particular, is a disease of the ID (Ehler et al. 2001; Perriard et al. 2003). These authors found increased expression of Nrap and, as expected with larger IDs, junctional proteins such as β-catenin. Another characteristic of these diseases is the movement of gap junctions to more lateral sites —– a return to a more embryonic situation (Severs et al. 2006). We have examined two examples of DCM in mice as well as in human tissue (Bennett 2015; Bennett et al. 2016; Wilson et al. 2014). The increase of disorder and size was apparent in all samples. Several observations suggested a loss of control of growth. Where measurable the amplitude of the ID folds was greater and the presence of filaments in the folds associated with sarcomere addition was observed more frequently (Wilson et al. 2014). There was also a loss of ID steps and an unbalanced insertion of sarcomeres over parts of IDs, which biased the direction of growth. DCM has been associated with the disruption of mitochondria organisation (van den Bosch et al. 2005; Wilding et al. 2006). In our observations, the differences were most pronounced near the ID (Bennett et al. 2016; Wilding et al. 2006; Wilson et al. 2014). Here, fewer mitochondria columns appeared to reach the ID, and in the extreme, several layers of sarcomeres could be seen adjacent to the ID without any accompanying mitochondria. These features suggest that, in DCM, coordinated growth of fibrils and mitochondria is compromised, and the balance of controlled contacts between the ID and the fibril /mitochondria /SR /T-tubule complex is lost.

Both of our mouse examples relate to dysfunction of proteins at the ID. In one, the cΔex3 mice, a β-catenin exon-conditional deletion leads to expression of a form of β-catenin which cannot be controlled by Wnt signalling or proteolysis (Hirschy et al. 2010). Consequent over accumulation of the protein leads to uncontrolled and catastrophic growth of AJs, hypertrophy, dilation and death. Our other example, the MLP KO, is less extreme in its phenotype (Arber et al. 1997). It has recently been shown that the role of MLP in heart is the inhibition of PKCα and that uninhibited PKCα activity at the ID in the absence of functional MLP leads to heart failure (Lange et al. 2016).

## Conclusions

The ID is a fascinating structure. It allows the cardiomyocytes to interact with several neighbouring cells and the complex structure of the heart to develop. It acts as tension transducer, structural prop, and multi signalling domain as well as a regulator of growth. Much of the analysis of its structures and functions described here has been obtained using data from high- resolution light and electron microscopy. No doubt, many other insights will emerge to interest us on our future journey on the waves of the ID.

## Abbreviations

ID: Intercalated disc
AJ: Adherens junction
TrJ: Transitional junction
AnkG: Ankyrin G
SASD: Spectrin-associated signalling domain
DCM: Dilated cardiomyopathy
NCX: Sodium calcium exchanger
SR: Sarcoplasmic reticulum
MLP: Muscle LIM protein
